# Demographics, Biochemical Characteristics, and Phases of Chronic Hepatitis B Virus Infection: Retrospective Analysis From a Secondary Care Setup

**DOI:** 10.7759/cureus.16558

**Published:** 2021-07-22

**Authors:** Muhammad Ali Khan, Muhammad S Haider, Baakh Nusrat, Syed Kumail Abbas Razvi, Zunaira Z Shah, Ali M Shah, Tahreem Khalid, Farhan Haleem

**Affiliations:** 1 Gastroenterology, Jinnah Postgraduate Medical Centre, Karachi, PAK; 2 Internal Medicine, Richmond University Medical Center, New York, USA; 3 Department of Medicine, Ziauddin University, Karachi, PAK; 4 Osteopathic Medicine, Sam Houston State University College of Osteopathic Medicine, Houston, USA

**Keywords:** demographics, hbv, biochemical paramenter, pakistan, hbv phases, frequency

## Abstract

Introduction

Hepatitis B virus (HBV) is the leading cause of cirrhosis in the developing world. Despite the development of effective vaccine(s) and direct-acting antivirals (DAAs) such as tenofovir and entecavir, the eradication of chronic HBV remains a distant dream in endemic regions. Factors such as treatment naivety, longer duration of disease, late diagnosis, family history of liver disorders and hepatocellular carcinoma, fatty liver disease, multiple comorbidities, alcoholism, use of tobacco products, noncompliance to drugs, and loss to follow-up all contribute to disease progression and development of complications.

In order to promote a better understanding of the treatment initiation, duration, and eventual outcomes, the European Association for the Study of the Liver (EASL) updated its guidelines in 2017 and introduced a new staging system for chronic HBV. Since these guidelines are relatively recent, data regarding the new staging is virtually absent in local/regional settings. Moreover, it has been observed that patients presenting to secondary care setups at major urban centers have disease characteristics quite distinct from those of their rural counterparts or patients presenting to tertiary care setups, even in the same cities. Additionally, there is scarce published data with regard to this aspect. With this study, we hope to make progress on all of those fronts.

Objective

We aimed to evaluate the demographics, biochemical characteristics, and phases of chronic HBV at the secondary care level.

Methods

This was a retrospective observational cohort conducted at the Sindh Government Hospital, Liaquatabad from January to December 2020. Patients of either gender who were aged ≥12 years, and diagnosed as a case of chronic HBV were eligible to be enrolled in the study. Only treatment-naive patients were enrolled in the study. Demographics, biochemical characteristics, and phases of HBV (in light of the updated guidelines issued by EASL in 2017 with respect to HBV) were recorded and analyzed. Patient confidentiality was ensured at all times.

Results

A total of 102 patients were enrolled in the study. The mean age of our cohort was 34.31 ±12.42 years, and the male-to-female ratio was 3:2. All referrals were made from within the city district. Housewives and laborers made up the majority of our patients. The mean alanine transaminase (ALT) levels were 39.83 ±21.33 IU/L; otherwise, the biochemical profile of the patients was unremarkable. Phase III was the most frequently recorded stage of chronic HBV; 41 (40.19%) of the patients were diagnosed with it. However, nearly half of the patients had chronic active hepatitis (phases II and IV). Only a single case each of hepatitis Delta virus and decompensated liver disease (DCLD) was recorded.

Conclusions

All patients of chronic HBV presenting at the secondary care level were referred locally, were relatively older, and exhibited a higher frequency of chronic active hepatitis (phases II and IV). This corresponded to borderline elevations of ALT. But the remainder of the biochemical profile was largely unremarkable due to the very low prevalence of cirrhosis among these patients.

## Introduction

Chronic liver disease, cirrhosis, and hepatocellular carcinoma (HCC) are all debilitating complications of chronic viral hepatitis, and the leading cause of these ailments in the developing world is the hepatitis B virus (HBV). Baruch Blumberg discovered HBV in 1965 and, by 1972, he had developed the first-ever vaccine for it as well [1,2]. Since then, great advancements have been made in the treatment and management of HBV, none more so important than the development of multiple direct-acting antivirals (DAAs) such as telbivudine, entecavir, and tenofovir [3]. DAAs are affordable and easy-to-administer oral medications that not only greatly reduce HBV activity but also prevent progression to cirrhosis and its complications [4,5]. Despite such remarkable breakthroughs, control and eradication of HBV in the developing world still remain elusive.

Although the global prevalence of HBV has declined in the last few decades, relatively higher rates of incidence and prevalence have been recorded in China, South Asia, and Africa [6]. Researchers have reported varying prevalence rates for HBV across Pakistan [7]; the World Health Organization (WHO) has listed Pakistan as an intermediate endemicity region with an HBV prevalence of 3-5% [6,8]. The estimated disease burden of HBV in Pakistan is between 8-12 million cases [9,10]. However, these numbers may not be accurate as data and records from secondary care setups, rural clinics, far-off dispensaries (usually suburban), and small private clinics and hospitals are all but missing.

These smaller centers only deal with a confined population in general, mostly residing within miles of the health facility. Therefore, the demographics and epidemiological and clinical characteristics of the patients presenting to these centers are vastly different from those recorded at the tertiary care level. Also, since these facilities are mostly community-based, they provide a better insight into the diseases and health-related issues specific to that area, which is not the case with major tertiary care centers that receive referrals from all over the country [11].

Local experience has shown that data from secondary care centers are notoriously difficult to preserve, chase down, or record. Lack of funding, unavailability of sufficient biochemical and radiological testing, loss to follow-up, and premature referrals all contribute to the flawed process because of which such valuable data goes missing or unpublished. However, we will not be exploring these factors in this study. Instead, we will be presenting data on an endemic disease, namely HBV, recorded from one of the largest secondary care setups in the city of Liaquatabad. We believe that this will not only add to the existing body of knowledge but also demonstrate the distinctiveness of data collected from smaller facilities.

## Materials and methods

We conducted an observational retrospective cohort study at the Sindh Government Hospital, Liaquatabad (SGHL) from January to December 2020. All data were acquired from the records register at the Gastroenterology and Hepatitis Clinic, SGHL. Patient confidentiality was ensured at all times. All biochemical and radiological testing was provided free of cost to the patients at the SGHL or other facilities of the social healthcare system. Patients of either gender who were aged ≥12 years, infected with HBV alone, and treatment-naive were eligible to be included in the study.

Hepatitis B surface antigen (HBsAg) serum assay was used initially for screening. Patients who tested positive (reactive) on these assays underwent further testing including liver function tests (LFTs), complete blood count (CBC), and polymerase chain reaction (PCR) quantitative analysis for HBV DNA. Assays for hepatitis B envelope antigen (HBeAg) and antibody (HBeAb), hepatitis Delta virus (HDV) antibodies (anti-Delta), PCR quantitative analysis for HDV, and ultrasound abdomen were also performed. Viral serologies for hepatitis C virus (HCV) were done as well. Chronic HBV was defined and classified into five distinct stages; these phases were defined in light of the most recent guidelines issued by the European Association for the Study of the Liver (EASL) on HBV [12]. These phases are as follows: HBeAg-positive chronic infection, HBeAg-positive chronic hepatitis, HBeAg-negative chronic infection, HBeAg-negative chronic hepatitis, and HBsAg-negative phase. For the purpose of this study, we did not use the old terminology. The phases are presented in Table 1.

**Table 1:** Definition of different phases of chronic hepatitis B virus as per the European Association for the Study of the Liver (EASL) guidelines issued in 2017 *As per guidelines issued by EASL in 2017 HBV: hepatitis B virus; HBeAg: hepatitis B envelope antigen; HBsAg: hepatitis B surface antigen: DNA: deoxyribonucleic acid: ALT: alanine transaminase

Phase	New name*	HBeAg status	Serum HBV DNA levels	ALT levels
Phase I	HBeAg-positive chronic infection	Positive	>10^7^ IU/ml	Normal
Phase II	HBeAg-positive chronic hepatitis	Positive	10^4^-10^7^ IU/ml	Elevated
Phase III	HBeAg-negative chronic infection	Negative	<2000 IU/ml	Normal
Phase IV	HBeAg-negative chronic hepatitis	Negative	>2000 IU/ml	Elevated
Phase V	HBsAg-negative phase	Negative	Undetectable	Normal

Patients with a positive HDV PCR result were labeled as chronic HDV cases. Patients with clinical evidence of stigmata of cirrhosis and decompensation (ascites, encephalopathy, variceal bleed, or coagulopathy) were designated as cases of decompensated liver disease (DCLD). Patients with concurrent HCV infection, chronic kidney disease, thyroid disorders, endocrinopathies, alcoholic liver disease, fatty liver disease, diabetes mellitus, solid organ malignancy (and/or on chemotherapy), immunocompromised patients, and patients with end-stage organ failure were excluded from the cohort. Patients with a history of the use of alternative or herbal or quack medications were also excluded from the study.

Data were analyzed using the SPSS Statistics software version 21.0 (IBM, Armonk, NY). The consecutive non-probability sampling technique was employed in our study. Mean and standard deviations were calculated for the quantitative variables of age, LFTs, and hematological profile. Frequency and percentages were calculated for the qualitative variables of gender, occupation, phases of chronic HBV, chronic HDV, and DCLD.

## Results

A total of 102 patients were enrolled in our study. The male-to-female ratio was 3:2. The mean age of our cohort was 34.31 ±12.42 years. Housewives and laborers made up the bulk of our patients. Every single referral was made from within the city district. Demographics of the patients enrolled in the study are presented in Table 2.

**Table 2:** Demographics of the patients enrolled in the study SD: standard deviation

Characteristics	Values (n=102)
Age, years, mean ±SD	34.31 ±12.42
Gender	
Male, n (%)	60 (58.82%)
Female, n (%)	42 (41.17%)
Occupation	
Housewife, n (%)	33 (32.35%)
Laborer, n (%)	33 (32.35%)
Student, n (%)	14 (13.72%)
Shopkeeper, n (%)	9 (8.82%)
Other, n (%)	9 (8.82%)
Unemployed, n (%)	4 (3.92%)

For the most part, the hematological profile and LFTs of the patients were unremarkable. Borderline elevations of serum alanine transaminase (ALT) levels were seen; normally, such elevations do not require further testing or strict follow-up, but with regard to chronic HBV, these are extremely significant. The biochemical profile of the patients is summarized in Table 3.

**Table 3:** Biochemical profile of the patients recorded in the study SD: standard deviation; ALT: alanine transaminase; AST: aspartate transaminase; GGT: gamma-glutamyl transferase; ALP: alkaline phosphatase

Variables	Values, mean ±SD (n=102)	Reference ranges
Hematological parameters		
Hemoglobin (gm/dl)	13.84 ±1.81	12.1-16.2
Mean corpuscular volume (fl)	86.44 ±5.93	78-95
White blood cell count (x 10^9^/L)	7.99 ±1.96	4-11
Platelets (x 10^9^/L)	265.77 ±65.94	150-450
Liver function tests		
ALT (IU/L)	39.83 ±21.33	0-41
AST (IU/L)	27.80 ±10.08	5-40
GGT (IU/L)	28.10 ±18.10	0-37
ALP (IU/L)	152.66 ±68.50	30-308 (adults)

Phase III, previously known as the inactive carrier phase, was the most frequently recorded phase in our study; approximately 40% of patients were diagnosed with HBeAg-negative chronic infection. However, nearly half of the patients were diagnosed with either phase II or phase IV of chronic HBV. Phases II and IV represent active hepatitis that requires treatment for long periods of time, and such a high number of cases of the active disease has not been previously recorded locally (see Discussion). The frequency of different phases of chronic HBV, HDV, and DCLD are shown in Table 4.

**Table 4:** Frequency of different phases of chronic hepatitis B virus, hepatitis Delta virus, and decompensated liver disease recorded in the cohort HBeAg: hepatitis B envelope antigen; HBsAg: hepatitis B surface antigen; HDV: hepatitis D virus

Phases	N (%)
Phase I (HBeAg-positive chronic infection)	9 (8.82%)
Phase II (HBeAg-positive chronic hepatitis)	18 (17.64%)
Phase III (HBeAg-negative chronic infection)	41 (40.19%)
Phase IV (HBeAg-negative chronic hepatitis)	32 (31.37%)
Phase V (HBsAg-negative phase)	Nil
Chronic HDV	1 (0.98%)
Decompensated liver disease	1 (0.98%)

## Discussion

HBV is endemic to Pakistan, but its disease characteristics do not seem to vary from province to province; Butt et al., Akhtar et al., Khan A et al., Awan et al., and Khan AU et al. have all previously described the demographic features, high prevalence rates, risk factors and high-risk groups, as well as treatment options and outcomes for HBV with fairly consistent results throughout [13-17]. The aforementioned studies have all featured a male predominance among HBV-infected patients and the mean age was between 20-40 years, which is comparable to our cohort. However, unlike previous studies, students did not make up a majority of our cohort. Housewives and laborers were the two most frequently encountered occupations. The low number of students registered might be due to shorter hours of functioning of secondary care outpatient departments that usually close before schools and colleges, or due to a lack of teaching institutes in the vicinity or improved vaccination status among the youth. The answer is onerous to pinpoint.

Local centers and secondary care setups treat a large number of gynecology and obstetrics (GYOB) patients in the developing world [18]. All housewives in our cohort were referred from the GYOB departments. This is due to improved screening protocols but also simultaneously underscores the need for improved surgical practices and the restriction of quackery, which are a major cause of HBV transmission in females and neonates in Pakistan [19]. Laborers included individuals involved in jobs that require hard manual work, such as electricians, carpenters, plumbers, construction workers, etc. The presence of a large number of laborers in the cohort was highly indicative of the socioeconomic status of the residents where our facility is located; it is our view that but for the ongoing coronavirus disease 2019 COVID-19 pandemic, many more laborers would have been enrolled in the study.

The new guidelines regarding HBV were expanded upon in 2017 by EASL; unsurprisingly, there is scarce data available with respect to the newly defined phases of HBV nationally. However, there have been multiple analyses documenting different stages of HBV based on HBeAg status and HBV DNA levels; Butt et al. and Hussain et al. have both reported high HBV inactive carrier (phase III) rates with relatively low rates of HBeAg-negative or -positive hepatitis (phases II and IV) [13,20]. While our inactive carrier (phase III) rate was comparable to previous local records, the rate of active hepatitis was inordinately high. Also, previous state and international reports have demonstrated a higher incidence and prevalence of HBeAg-positive chronic hepatitis compared to HBeAg-negative chronic hepatitis in controls [21,22]; we observed a reversal of this phenomenon in our cohort.

One of the reasons for such a high rate of chronic active hepatitis could be related to the high number of referrals from GYOB; these women were either being screened for an elective cesarean section or as part of the now mandatory screening program for all childbearing women. Qureshi et al. have illustrated an HBeAg positivity rate of 22% [23], which corresponds to our results, and comparable rates of HBeAg positivity in pregnant women were also reported by Wen et al. [24]. Other variables including a high number of middle-aged patients (mostly laborers), extensive duration of the disease, and treatment naivety might have also contributed to the disproportionate rate of chronic active hepatitis. We did not evaluate smoking habits and the use of tobacco products or other recreational substances, which are fairly common in the locale. These deleterious habits definitely have a negative impact on disease progression, and a more detailed analysis is required to objectively identify their role in chronic HBV.

We only recorded borderline elevations of ALT in our study; borderline elevations are defined by the American College of Gastroenterology and EASL as <2x the upper limit of normal [12,25]. These borderline elevations, while harmless in normal healthy adults, confer considerable risk for disease progression and development of complications such as cirrhosis and HCC in HBV-infected patients [12]. The mean ALT value for phases II and IV alone was 57.28 ±21.95 IU/L; phases II and IV are characterized by elevated ALT levels and it was unsurprising to observe such values. The remainder of the biochemical profile was unremarkable, in part due to the near-absence of decompensation in our patients.

Phase II is treated with DAAs for a minimum period of 48 weeks; the eventual goal is either the loss of HBsAg or undetectable levels of HBV DNA [12]. Even once these goals are achieved, patients have to continue with pharmacotherapy for a consolidation period of 6-12 months [12]. It is not uncommon to find overlap or transitions into other phases of chronic HBV once treatment is initiated for phase II. Unlike phase II, there are no clear-cut endpoints for the treatment of phase IV; as such, it usually requires lifelong treatment with DAAs [12]. Guidelines recommend surveillance of patients with chronic HBV irrespective of treatment outcomes with annual or biannual abdominal ultrasound and LFTs [12].

The risk of progression to cirrhosis without treatment in HBeAg-positive hepatitis is 4-7% per year, and with HBeAg-negative infection, it is 2-3% per year [26]. Approximately half of our controls had active chronic hepatitis and all were treatment-naive, yet we only recorded a single case of DCLD. Even with our small sample size, this was an unexpected finding. To us, this was suggestive of the fact that patients from large urban centers such as ours have easier and timelier access to modern medical expertise. There are a lot of social welfare organizations in the city that provide assistance to patients with respect to healthcare and economic matters, but such centers are not always present in rural or peripheral centers. Corporations, independent contractors, small business owners, and educational institutes also make it mandatory for their staff/students to get screened for chronic hepatitis at regular intervals; thanks to periodic screening, patients are more likely to be diagnosed at earlier stages of chronic HBV. All of this adds up to higher rates of chronic active hepatitis without cirrhosis, which is amenable to pharmacological therapy.

The actual prevalence of chronic HDV in Pakistan is still uncertain. Researchers have previously described prevalence rates ranging from 4-67%; these variations were seen with respect to age, gender, provinces, occupation, and HDV genotype [13,17,27]. The highest prevalence of HDV has been reported in the province of Sindh [17]. An incidence rate of 30.8% was reported by Aftab et al. in pregnant HBsAg-positive women; however, the overall prevalence rate of HDV in all controls was <1% [26]. It is highly unlikely that the actual prevalence of chronic HDV is <1% in a major district of Karachi, Sindh. Our results are more likely a reflection of a smaller sample size notably affected by the ongoing COVID-19 pandemic.

Not a single case of HBsAg-negative phase (phase V) was encountered in our cohort. Also known as occult HBV infection or HBsAg clearance phase previously, it is a rarely encountered condition. Phase V represents the optimal outcome in patients infected with chronic HBV. The reported frequency of this phase even in subjects on treatment is extremely low. In their study, Buti et al. did not record the loss of HBsAg at all in patients treated with either tenofovir or entecavir for 48 weeks [28]. Charlton et al. and Aziz et al. have reported analogous outcomes [29,30]. It was very unlikely that we would see a case of phase-V chronic HBV in such a small sample size where all patients were treatment-naive.

Limitations of the study

We did not perform a liver biopsy on any of our patients. Similarly, transient elastography was not done at all. The sample size was too small for such procedures; this study was held during the COVID-19 pandemic, and hence loss to follow-up was very high, and this was the primary reason that we did not evaluate or record treatment options or outcomes in our patients.

## Conclusions

Based on our findings, patients infected with chronic HBV who present at the secondary care level exhibited much higher frequencies of chronic active hepatitis compared to their counterparts at rural and tertiary care setups. All patients at such setups were referred locally (from within the city district). Despite being middle-aged or older and treatment-naive at the time of diagnosis, a vast majority of the patients did not have any biochemical or clinical evidence of cirrhosis. This was most likely due to patients either seeking prompt medical help or being screened earlier in their disease course; this timely referral was made possible due to better healthcare policies and facilities available to the residents of urban centers even at the secondary care level.
